# Small dense low-density lipoprotein cholesterol is strongly associated with NIHSS score and intracranial arterial calcification in acute ischemic stroke subjects

**DOI:** 10.1038/s41598-020-64715-9

**Published:** 2020-05-06

**Authors:** Tao Yao, Qi Long, Jing Li, Gang Li, Yanbin Ding, Qin Cui, Zhichao Liu

**Affiliations:** 1 Department of Neurology, Wuhan University, Renmin Hospital, Wuhan, China; 2 Emergency Department, Hubei Provincial Hospital of Traditional Chinese Medicine, Wuhan, 430061 China; 3 Hubei Province Academy of Traditional Chinese Medicine, Wuhan, China; 4 Department of Neurology, Hubei Provincial Hospital of Traditional Chinese Medicine, Wuhan, 430061 China

**Keywords:** Stroke, Risk factors

## Abstract

Intracranial artery calcification (IAC) is an important risk factor for cerebral infarction and a key biomarker for intracranial artery stenosis. Small dense low-density lipoprotein cholesterol (sd-LDL-c) was independently associated with increased cardiovascular events and coronary calcification. Our study assessed whether sd-LDL-c is an independent factor for IAC in acute ischemic stroke (AIS) patients. This cross-sectional study involved a total of 754 patients with AIS (mean age: 65 ± 13.2 years). All the patients had received brain computed tomography angiography (CTA) examination to evaluate IAC. Serum sd-LDL-c levels and other biochemical parameters were analyzed. Admission NIHSS score and mRS score at discharge were collected. After 60-days 85 patients died during hospitalization and follow-up. Partial correlation analysis showed that serum sd-LDL-c levels were associated with admission NIHSS score and IAC score after adjusted age and gender. Logistic regression analysis showed that serum sd-LDL-c levels independently predicted NIHSS scores (β = 1.537, 95%CI: 0.134-2.878, p = 0.042) and IAC scores (β = 1.355, 95%CI: 0.319-2.446, p = 0.015). The average level of sd-LDL-c in patients who died was also significantly increased compared to survival patients (1.04 ± 0.59 vs 0.88 ± 0.44 mmol/L, p = 0.017). However, multivariate logistic regression analysis showed serum sd-LDL-c levels could not predict all-cause mortality and prognosis in AIS patients. Our study found that sd-LDL-c as a strong atherogenic lipid particle can independently predict admission NIHSS scores and the severity of cerebral artery calcification in AIS patients. However, its prognostic value in AIS patients still needs further study in the future.

## Introduction

It has been reported that arterial calcification is a passive process of calcium deposition in cells and tissues due to an imbalance in the calcium and phosphorus metabolism in the body^1,2^. As a degenerative disease that progresses with aging, arterial calcification was considered as a sign of vascular aging. However, recent studies have shown that arterial calcification is a complex process with multiple etiologies, pathways and mechanisms^3–5^. Arterial calcification can be regarded as a biological phenomenon. The degree of arterial calcification is related to the burden of an atherosclerotic plaque^6^. Moreover, there is a significant correlation between lipid metabolism disorders and coronary artery calcification^7^.

Intracranial arterial calcification (IAC) is associated with ischemic stroke, cognitive decline and other vascular events^8^. IAC is an important risk factor for cerebral infarction and an important marker of intracranial artery stenosis. The result of the Rotterdam study showed that IAC is a major risk factor for stroke in white people^9^. The strong atherogenic lipid component small dense low-density lipoprotein cholesterol (sd-LDL-c) is strongly associated with coronary artery calcification in healthy women and asymptomatic adults at intermediate risk of cardiovascular disease^10,11^. However, the relationship between sd-LDL-c and IAC has not been evaluated in general and more specific, in acute ischemic stroke (AIS) patients.

Zeljkovic *et al*. showed that increased sd-LDL-c level was related to increased morbidity and mortality in AIS^12^. However, Markaki *et al*. found that high cholesterol levels are associated with improved long-term prognosis in AIS patients^13^. The National Institutes of Health Stroke Scale (NIHSS) score reflects the severity of an acute cerebral infarction and is closely related to the prognosis of patients. In AIS patients, the relation of sd-LDL-c and other lipid components with NIHSS score at admission is not clear. Therefore, we designed this study and evaluated the relationship between sd-LDL-c and IAC in AIS patients. Furthermore, it was examined whether sd-LDL-c can independently predict NIHSS score and short-term prognosis in AIS patients.

## Materials and Methods

### Subjects

This cross-sectional study was performed at the Department of Neurology of the Renmin Hospital, Wuhan University in China. The present study received approval by the Institutional Review Board of the University Affiliated Hospital. All patients referred for AIS between January 2017 and December 2018 were screened. Patients underwent brain CT and/or magnetic resonance imaging (MRI), and the diagnosis of ischemic stroke was based on the consensus of at least two neurologists. The present study complied with the Declaration of Helsinki and was approved by the Human Research Ethics Committee of Renmin Hospital of Wuhan University (Wuhan, China, No. 2016H11018). Written informed consent was obtained from each participant. We had signed consent from every participant to participate in the study. The results are all anonymous. No individual result is presented and only group statistics were provided.

According to AHA/ASA stroke guidelines^14^, 913 patients with initial diagnosis of AIS were admitted to the Wuhan University affiliated Renmin Hospital. These patients were enrolled in the study: 18-85 years old, duration of onset of symptoms to hospital less than 48 hours, patients or their guardians signed the informed consent. Other types of strokes including transient ischemic attack and hemorrhagic stroke were excluded. 159 patients were excluded from the study: 30 patients did not undergo computed tomography angiography (CTA) examination, 63 patients had missing follow-up results, 31 patients had a history of chronic liver disease and abnormal liver function, and 35 patients had no NIHSS score at admission. Meanwhile, in order to eliminate the effect of drugs on lipid metabolism, we further excluded 96 statin users and using other lipid-lowering agents of 26 patients. A total of 632 patients were included in logistic regression analysis. The primary outcome was 60-day mortality after admitted to hospital.

### Biochemical measurements

All patients included in the study underwent head CT, electrocardiogram (ECG), MRI, transthoracic echocardiography (TEE) and had standard laboratory examinations at admission. Blood samples were obtained from an antecubital vein and collected in vacutainer tubes containing EDTA. Patients were asked to fast for eight hours before blood draw, and liver enzyme levels, serum lipid profiles including total triglyceride (TG), total cholesterol (TC), low-density lipoprotein cholesterol (LDL-c) and high-density lipoprotein cholesterol (HDL-c), biochemical parameters of the kidney, fasting plasma glucose (FPG), homocysteine (Hcys) and glycosylated hemoglobin (HbA1c) levels were determined on an Olympus AU600 analyzer (Olympus, Tokyo, Japan). sd-LDL-c was measured by the method of Hirano *et al.*^15^ with minor modification^16^ using the commercially available assay kit (sd-LDL-c SEIKEN, Denka Seiken Co., Ltd, Tokyo, Japan). Apolipoproteins (apo) A-1, and B were measured by immunoturbidimetry (Daiichi Pure Chemicals Co., Ltd., Tokyo, Japan). The following variables were collected in the acute phase of ischemic stroke: age, gender, cause of ischemic stroke (according to TOAST criteria)^17^, and confirmed or new detected risk factors such as hypertension, diabetes mellitus (DM), coronary heart disease, smoking, long-term alcohol consumption, atrial fibrillation (AF) and BMI. Hyperlipidemia was defined as serum TC was over 5.2 mmol/L or/and serum TG was over 1.7 mmol/L. Those with hyperlipidemia were subgrouped into combined hyperlipidemia, hypertriglyceridemia, and hypercholesterolemia. Combined hyperlipidemia was defined as both serum TC was over 5.2 mmol/L and serum TG was over 1.7 mmol/L; hypertriglyceridemia was defined as serum TG was over 1.7 mmol/L; hypercholesterolemia was defined as serum TC was over 5.2 mmol/L.

### Assessment of IAC score

All patients underwent multidetector brain CTA using a 64-slice spiral CT device (GE Healthcare, Milwaukee, WI, USA) with the following parameters:120 kVp, 140 mA, 0.9-mm section thickness, 0.9-mm slice acquisition interval, and intravenous administration of 80 mL of iohexol at a rate of 5.0 mL/s. Bone window CT images covered the whole brain (from the skull base to the vertex) to identify IAC. IAC foci were defined as hyperdense foci with a median density greater than 130 Hounsfield units. We used the 5-point scale semi-quantitative scoring system proposed by Babiarz *et al*. to evaluate IAC^18^. The highest calcification score for each cerebral artery was selected and the calcification score of all evaluated arteries were added to obtain the patient’s total calcification score. The severity of IAC was graded based on the total calcification score: 0 was absent, 1–4 was mild, 5–8 was moderate, and 9–12 was severe^19^. Two experienced neurologists independently reviewed the images from CT angiography and graded the degree of cerebral artery calcification.

### Statistical analyses

SPSS version 22.0 (SPSS Inc, Chicago, IL, USA) was used for statistical analysis. Continuous variables with a normal distribution were compared using the Student’s t-test and ANOVA. Categorical variables were analyzed using the chi-squared test. Patients were divided in tertiles based on their serum sd-LDL-c levels. Partial Spearman correlation coefficients were used to examine the association between serum sd-LDL-c with IAC scores and admission NIHSS scores after adjustment for age and gender. To assess the association between serum sd-LDL-c and severe IAC, binary logistic regression analysis was used. Further analyses were performed to calculate multivariable-adjusted ORs (95% CIs) of severe IAC for the highest tertile versus the lowest tertile of serum sd-LDL-c levels after adjustment for covariates. To determine whether sd-LDL-c and other cholesterol components or ratio predicted short-term all-cause mortality risk of AIS patients, Cox regression model was applied after adjustment for the covariates. Differences were considered significant when *P* < 0.05.

## Result

A total of 754 AIS patients, with an average age of 65 ± 13.2years, including 515 males were enrolled in this study. After 60-days 85 patients died during hospitalization and follow-up. These patients included 632 hypolipemic drugs users and 122 non-hypolipemic drugs users. The average sd-LDL-c level of all patients was 0.90 ± 0.46 mmol/L. There was no difference of sd-LDL-c level between hypolipemic drugs users and non-hypolipemic drugs users (0.89 ± 0.47 *vs* 1.07 ± 0.37 mmol/L, p = 0.067). We divided non-hypolipemic drugs users into tertiles (0.12-0.64 mmol/L, 0.65-1.07 mmol/L, > 1.07 mmol/L) based on their sd-LDL-c level. Analysis of variance showed that the NIHSS score, IAC score and mortality rate in the highest tertile of sd-LDL-c were significantly higher than in the other two tertiles (Table 1). The average level of sd-LDL-c in all patients who died was also significantly increased compared to survival patients (1.04 ± 0.59 vs 0.88 ± 0.44 mmol/L, P = 0.017).

**Table 1:** Clinical and laboratory data for AIS subjects divided according to their sd-LDL-c levels and hypolipemic drugs using.

sd-LDL-C	Tertiles1, n = 211 ≤ 0.64 mmol/L	Tertiles 2,n = 215 0.65-1.07 mmol/L	Tertiles 3,n = 206 ≥1.07 mmol/L	*p* value	Non-hypolipemic drugs users, n = 632	Hypolipemic drugs users, n = 122	All patients, n = 754	*p* value
Age(years)	69.1 ± 13.6	66.2 ± 12.0	61.7 ± 13.0	<0.001	64.3 ± 13.2	70.2 ± 10.6	65.3 ± 13.1	0.001
BMI(kg/m^2^)	22.81 ± 2.50	23.22 ± 2.61	23.44 ± 2.79	0.320	23.17 ± 2.49	23.38 ± 2.56	23.27 ± 2.52	0.335
Smoking,n(%)	66 (31.3%)	71 (33.0%)	78 (7.8%)	0.340	237 (37.5%)	30 (24.3%)	267 (35.4%)	0.022
Drinking, n(%)	40 (19.0%)	43 (20.0%)	53 (25.7%)	0.194	152 (24.1%)	18 (14.3%)	170 (22.6%)	0.045
HT,n(%)	136 (64.4%)	155 (72.1%)	135 (65.5%)	0.191	413 (65.4%)	105 (85.7%)	518 (68.6%)	<0.001
DM, n(%)	56 (26.5%)	75 (34.9%)	76 (36.9%)	0.057	192 (30.4%)	37 (30.0%)	229 (30.3%)	0.538
CHD, n(%)	14 (6.6%)	20 (9.3%)	22 (10.7%)	0.335	44 (7.0%)	40 (32.9%)	84 (11.1%)	<0.001
Hyperlipidemia,n(%)	55 (26.1%)	70 (32.6%)	106 (51.5%)	<0.001	154 (24.4%)	99 (81.4%)	253 (33.5%)	<0.001
AF, n(%)	20 (9.4%)	26 (12.1%)	30 (14.6%)	0.280	72 (11.4%)	19 (15.7%)	91 (12.0%)	0.201
SBP,mmHg	149.8 ± 26.3	149.7 ± 24.7	152.9 ± 26.5	0.597	149.2 ± 26.1	155.1 ± 27.7	150.3 ± 26.1	0.088
DBP, mmHg	81.8 ± 13.6	83.2 ± 15.1	88.8 ± 15.9	0.05	84.7 ± 15.5	85.5 ± 15.1	85.0 ± 15.3	0.680
Cr (umol/L)	77.3 ± 28.4	76.8 ± 29.1	78.2 ± 28.6	0.881	76.1 ± 29.8	80.5 ± 30.7	76.4 ± 29.1	0.269
UA (umol/L)	345.6 ± 105.2	368.9 ± 120.5	392.7 ± 120.1	0.005	369.6 ± 121.1	370.8 ± 112.8	369.7 ± 117.5	0.938
TC (mmol/L)	3.96 ± 0.95	4.51 ± 0.89	5.11 ± 0.96	<0.001	5.09 ± 1.16	4.39 ± 0.99	4.62 ± 1.05	<0.001
TG (mmol/L)	1.28 ± 0.69	1.63 ± 1.40	2.25 ± 1.49	<0.001	2.11 ± 1.88	1.60 ± 1.13	1.69 ± 1.30	0.002
HDL-C (mmol/L)	1.20 ± 0.65	1.07 ± 0.33	1.14 ± 0.39	0.105	1.13 ± 0.64	1.14 ± 0.40	1.13 ± 0.59	0.883
LDL-C (mmol/L)	2.21 ± 0.93	2.61 ± 0.71	2.96 ± 0.85	<0.001	2.87 ± 1.00	2.52 ± 0.82	2.77 ± 0.85	0.002
ApoA1 (g/L)	1.30 ± 0.22	1.33 ± 0.25	1.29 ± 0.23	0.787	1.31 ± 0.21	1.33 ± 0.26	1.31 ± 0.22	0.612
ApoB (g/L)	0.70 ± 0.17	0.91 ± 0.24	1.06 ± 0.22	<0.001	0.90 ± 0.25	0.99 ± 0.25	0.91 ± 0.25	0.008
TC/HDL	3.66 ± 0.95	4.27 ± 1.35	5.12 ± 1.44	<0.001	4.78 ± 1.69	4.28 ± 1.33	4.33 ± 1.38	0.006
TG/HDL	1.22 ± 0.81	1.65 ± 1.60	2.29 ± 1.70	<0.001	2.15 ± 1.28	1.63 ± 1.32	1.72 ± 1.51	0.009
ApoB/ ApoA1	0.54 ± 0.13	0.70 ± 0.20	0.85 ± 0.27	<0.001	0.70 ± 0.22	0.78 ± 0.35	0.72 ± 0.25	0.023
HbA1c (%)	6.70 ± 1.60	6.76 ± 1.63	7.42 ± 1.59	0.110	7.06 ± 1.48	7.00 ± 1.84	7.04 ± 1.63	0.919
D-dimer (mg/L)	1.22 ± 2.01	1.35 ± 2.38	1.27 ± 2.56	0.953	1.25 ± 2.40	1.60 ± 2.48	1.25 ± 2.33	0.276
Hcys (umol/L)	20.22 ± 12.39	21.87 ± 16.31	16.52 ± 8.94	0.05	19.17 ± 12.64	18.54 ± 10.21	19.1 ± 12.4	0.723
IAC score	3.36 ± 3.23	3.57 ± 3.41	4.45 ± 3.78	0.01	3.65 ± 3.46	4.82 ± 3.77	3.83 ± 3.50	0.01
Death,n (%)	20 (9.5%)	23 (10.7%)	29 (14.1%)	0.310	72 (11.6%)	13 (10.7%)	85 (11.3%)	0.324
NIHSS score	5.11 ± 5.05	6.67 ± 6.10	7.89 ± 6.68	<0.001	6.31 ± 6.12	8.25 ± 7.39	6.55 ± 6.34	0.019
mRS score	2.13 ± 1.64	2.30 ± 1.79	2.50 ± 1.81	0.225	2.30 ± 1.82	2.66 ± 1.83	2.34 ± 1.82	0.134
Severe IAC, n (%)	22 (10.4%)	30 (13.9%)	50 (24.3%)	<0.001	85 (13.4%)	32 (26.2%)	117 (15.5%)	0.001

AF, atrial fibrillation; BMI, body mass index; SBP, systolic blood pressure; DBP, dilated blood pressure; CHD, coronary heart disease; DM, diabetes mellitus; HbA1c, glycated hemoglobin; LDL-C, low-density lipoprotein cholesterol; HDL-C, high-density lipoprotein cholesterol; TC, total cholesterol; TG, total triglyceride; UA, uric acid; Apo B: Apolipoproteins B; ApoA-1: Apolipoproteins A-1; sd-LDL-C: Small dense low-density lipoprotein cholesterol; HT, hypertension; Hcys, homocysteine; IAC, intracranial artery calcification; mRS score, Modified Rankin Scale score; NIHSS, National Institutes of Health Stroke Scale.

**Figure 1:**
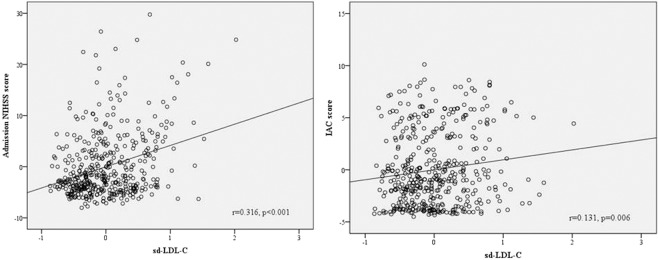
Partial Spearman correlation coefficients showed the association between serum sd-LDL-c levels with admission NIHSS score and IAC score after adjustment for age and gender.

We analyzed the correlation between sd-LDL-c level and IAC score and admission NIHSS score in non-hypolipemic drugs users. After adjusting for age and sex, partial correlation analysis showed that sd-LDL-c was correlated with IAC score (r = 0.131, P = 0.006) and admission NIHSS score (r = 0.316, P < 0.001, Fig. 1).

Univariate linear logistic regression analysis showed that the level of sd-LDL-c predicted admission NIHSS score (β = 2.248, 95%CI: 1.136-3.519, p < 0.001)and IAC score (β = 1.209, 95%CI: 0.498-1.766, p < 0.001). After adjusting for the variables, sd-LDL-c was still independently correlated with admission NIHSS score (β = 1.537, 95%CI: 0.134-2.878, p = 0.042, Table 2) and IAC score (β = 1.355, 95%CI: 0.319-2.446, p = 0.015, Table 2). Cox logistic regression analysis showed that sd-LDL-c was associated with short-term mortality risk in AIS patients without adjusting for variables (OR = 1.936, 95%CI: 1.134-3.306, p = 0.016, Table 2). However, after adjustment for DBP, HbA1c, admission NIHSS score, and infarction type, etc., sd-LDL-c was no longer an independent predictor of the short-term mortality in AIS patients (Table 2).

**Table 2:** Correlations of sd-LDL-C with NIHSS score, IAC score and death.

NIHSS score	β	95%CI	*p* value
Unadjusted	2.248	1.136-3.519	<0.001
Model 1	2.335	1.209-3.621	<0.001
Model 2a	2.478	1.218-3.924	<0.001
Model 3a	1.537	0.134-2.878	0.042
**IAC score**	**β**	**95%CI**	***p*** **value**
Unadjusted	1.209	0.498-1.766	<0.001
Model 1	1.051	0.387-1.688	0.005
Model 2a	1.403	0.611-2.335	<0.001
Model 3a	1.355	0.319-2.446	0.015
**Death**	**OR**	**95%CI**	***p*** **value**
Unadjusted	1.936	1.134-3.306	0.016
Model 1	2.078	1.188-3.636	0.01
Model 2b	1.109	0.460-2.676	0.817
Model 3b	0.608	0.202-1.828	0.376

Model 1: adjusted for age, gender, smoking, HT, DM, AF;

Model 2a: Model 1 + TC, TG, LDL-C, HDL-C;

Model 3a: Model 2a + HbA1c, Hcys, DBP and UA;

Model 2b: Model 1 + DBP, Cr, HbA1c and UA;

Model 3b: Model 2b + D-dimer, admission NIHSS score, Hcys and infarction type.

**Figure 2:**
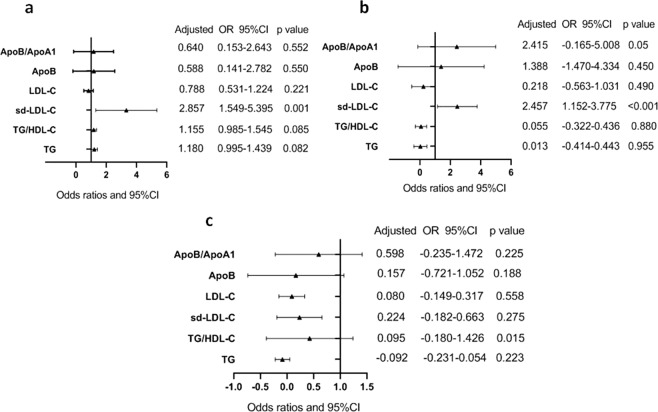
Fully adjusted multivariable logistic regression models. (**a**) Six lipid components or ratios to predict sever IAC; (**b**) to predict admission NIHSS score; (**c**) to predict mRS score at discharge. Adjusted for age, sex, hypertension, DM, AF, HbA1c.

We defined patients with an IAC score above 4 as having severe calcification. The level of sd-LDL-c in patients with severe IAC was significantly increased compared to non-severe IAC patients (1.09 ± 0.55 vs 0.86 ± 0.44 mmol/L, P < 0.001). However, there was no significant difference in admission NIHSS score (6.67 ± 5.78 *vs* 6.53 ± 6.45, p = 0.873), mortality rate (8.1% *vs* 12.4%, p = 0.198) between severe intracranial calcification patients and non-severe calcification patients. We assessed the predictive value of TG, TG/HDL-c, sd-LDL-c, LDL-c, ApoB and ApoB/ApoA1 for severe intracranial calcification, admission NIHSS score and mRS score at discharge. After adjusting for age, sex, hypertension, DM, AF, HbA1c variables and so on, logistic regression analysis showed that only sd-LDL-c predicted the severity of IAC (OR = 2.857, 95%CI: 1.549-5.395, p = 0.001, Fig. 2a). However, there was no significant difference in the risk of severe calcification between tertiles groups.

In addition, the predictive value of six lipid components or cholesterol ratios for admission NIHSS scores and mRS scores at discharge were assessed. Similarly, sd-LDL-c (OR = 2.457, 95% CI: 1.152-3.775, p < 0.001, Fig. 2b) and ApoB/ApoA1 (OR = 2.415, 95% CI: -0.165-5.008, p = 0.05, Fig. 2b) independently predicted admission NIHSS score after adjusting for relevant variables. However, only TG/HDL-c (OR = 0.095, 95% CI: -0.180-1.426, p = 0.015, Fig. 2c) independently predicted the mRS score at discharge.

## Discussion

In this study, we assessed the relationship between sd-LDL-c levels and IAC, NIHSS scores, short-term all-cause mortality and mRS scores at discharge in AIS patients. The level of sd-LDL-c in patients who died and those with severe IAC were significantly increased. Although there seemed to be a relation between sd-LDL-c and mortality risk and mRS score, sd-LDL-c did not independently predict all-cause mortality and prognosis. sd-LDL-c independently predicted the NIHSS score and IAC score in AIS patients, but there was no significant difference in IAC in patients with different levels of sd-LDL-c.

As a marker of intracranial atherosclerosis, IAC is an easily measurable and promising biomarker reflecting the severity of intracranial vascular disease. Chen *et al*. were the first to demonstrate that the incidence of IAC was higher in Chinese patients with ischemic stroke in a cross-sectional study^20^. In a Danish population, Ovesen *et al*. showed that the severity of intracranial atherosclerosis detected during emergency assessment (graded by the number of cerebral artery calcification) predicts an increased risk of stroke recurrence^21^. The Rotterdam study quantified the volume of IICA calcification measured by CT scan, and determined that IAC was the main risk factor for stroke in white people^9^. Although IAC is often observed, there is currently lack of a standard method to quantify the severity of IAC. Therefore, using severity of IAC in clinical practice is limited.

In our study subjects, sd-LDL-c can independently predict the severity of IAC. sd-LDL-c, as a lipid particle, can easily enter the arterial wall due to its small size. It binds to glycoproteins in the arterial wall and causes oxidative and inflammatory reactions, thereby damaging the intima of blood vessels^22^. In previous studies, we found that sd-LDL-c can independently predict the progression of arteriosclerosis^23^. At the present study, although the cholesterol levels of AIS patients who used lipid-lowering drugs before admission was lower than that of those who did not, there was no difference in sd-LDL-c level between them. The age, IAC score and the proportion of severe IAC were significantly higher in patients who used lipid-lowering drugs than those who did not. It has been suggested that statins may increase the proportion of calcium in coronary atherosclerotic plaque^24^.Simultaneously, a meta-analysis also showed that lowering LDL had no inhibitory effect on coronary artery calcification^25^.We found that LDL could not independently predict the degree of IAC. These information suggest that lowering traditional cholesterol level and inhibiting vascular calcification may be two different pathophysiological mechanisms.

Hyperlipidemia is a well-known risk factor for cardiovascular disease. However, findings of previous observational studies attempting to explain the effects of lipid levels on the prognosis of stroke are variable^13,26,27^. Large artery atherosclerosis is the main subtype of cerebral infarction in the TOAST classification system. However, the proportion of cerebral infarction caused by large artery atherosclerosis in different populations varies^28^. In our study population, the level of sd-LDL-c in patients who had died was significantly increased. Although sd-LDL-c is correlated with mortality risk and mRS score at discharge, it was not an independent predictor of all-cause mortality and short-term prognosis. sd-LDL-c can independently predict the NIHSS score at admission. Zeljkovic *et al*. conducted a study with a short-term follow-up of 200 patients with new onset of cerebral infarction and found that sd-LDL-c independently predicted the occurrence and short-term mortality of AIS^12^. Our study showed a different result. This may be explained by different subtypes of cerebral infarction in the study population and the length of follow-up.

The NIHSS score has been widely used in evaluating the severity of stroke in patients. TG, LDL-c, HDL-c, TG/HDL, ApoB and other lipid components or proportions are to some degree correlated with the NIHSS score^29–31^. Yasuhiro *et al*. semi-quantitatively detected sd-LDL-c in peripheral blood of new diagnosed AIS patients^32^. The results showed that sd-LDL-c was associated with adverse prognosis and mRS scores. However, sd-LDL-c was not related to the NIHSS score. We quantitatively measured sd-LDL-c and found that it was independently correlated with the NIHSS score. sd-LDL-c is a strong atherogenic factor and can penetrate into the arterial wall more easily and cause oxidative stress, which can lead to the onset of AIS and aggravate the degree of an infraction.

This study has the following limitations. First, the short follow-up of only 60 days affects the accuracy of sd-LDL-c in predicting prognosis. Second, up to now, there is no accurate and widely accepted quantitative measurement for IAC score. As in other study methods, IAC was semi-quantitatively determined and graded in our study. Third, the number of patients included in the study is small. The relationship between sd-LDL-c level and IAC, NIHSS scores and prognosis in AIS patients needs to be examined in large-scale prospective studies.

## Conclusion

In conclusion, we systematically evaluated the relationship between serum sd-LDL-c levels and other lipid components or ratios with IAC score and admission NIHSS score in the Chinese AIS patients. sd-LDL-c as a strong atherogenic lipid particle is demonstrated that could independently predict admission NIHSS scores and the severity of cerebral artery calcification at the present study. However, its prognostic value in AIS patients still needs further study in the future.

### Ethics approval and consent to participate

This study complied with the Declaration of Helsinki and was approved by the Human Research Ethics Committee of Renmin Hospital of Wuhan University (Wuhan, China, No.2016H11018).
